# Supplementary material for: Dinucleoside polyphosphates act as 5′-RNA caps in bacteria
Source: Nat Commun. 2020 Feb 26;11:1052. doi: 10.1038/s41467-020-14896-8 (PMC7044304; doi:10.1038/s41467-020-14896-8)
Supplement: Supplementary file 1 — Supplementary Information [file 41467_2020_14896_MOESM1_ESM.pdf]

Supplementary Information

Dinucleoside polyphosphates act as 5'-RNA caps in bacteria

Oldřich Hudeček[1]*, Roberto Benoni[1]*, Paul E. Reyes-Gutierrez[1], Martin Culka[1], Hana Šanderová[2], Martin Hubálek[1], Lubomír Rulíšek[1], Josef Cvačka[1], Libor Krásný[2], Hana Cahová[1]

Supplementary Table 1: Names and sequences of oligonucleotides and plasmids

Name	Sequence
35A	5’-CAGTAATACGACTCACTATT**A**GGGAAGCGGGCATGCGGCCAGCCATAGCCGATCA-3’
35G	5’-CAGTAATACGACTCACTATA**G**GGGAAGCGGGCATGCGGCCAGCCATAGCCGATCA-3’
Plasmid 458	5’-GAATTCGCGGTCAGAAAATTATTTTAAATTTCCTCTTGTCAGGCCGGAATAACTCCCTATAATGCGCCACCACTGACACG**A**AGCTTGGGTCCCACCTGACCCCATGCCGAACTCAGAAGTGAAACGCCGTAGCGCCGATGGTAGTGTGGGGTCTCCCCATGCGAGAGTAGGGAACTGCCAGGCATCAAATAAAACGAAAGGCTCAGTCGAAAGACTGGGCCTTT-3’
FV21	5’-AGACACGACTTATCGCCACTGGCAGCAGCCACTGGTAACAGGATTAGCAGAGCGAGGTATGTAGGCGGTGCTACAGAGTTCTTGAAGTGGTGGCCTAACTACGGCTACACTAGAAGG**A**CAGTATTTGGTATCT-GCGCTCTGCTGAAGCCAGTTACCTTCGGAAAAAGAGTTGGTAGCTCTTGATCCGG-3’
FV21fwd	5’-AGACACGACTTATCGCCAC-3’
FV21rev	5’-CCGGATCAAGAGCTACCAAC-3’
#3071	5’-GGAATTCCATATGGCGACATACCTTATTGG-3’
#3072	5’-CCGCTCGAGAGACGCCGCCGCTTCGCCCA-3’
2ntG	5’-CAGTAATACGACTCACTATA**G**G-3’

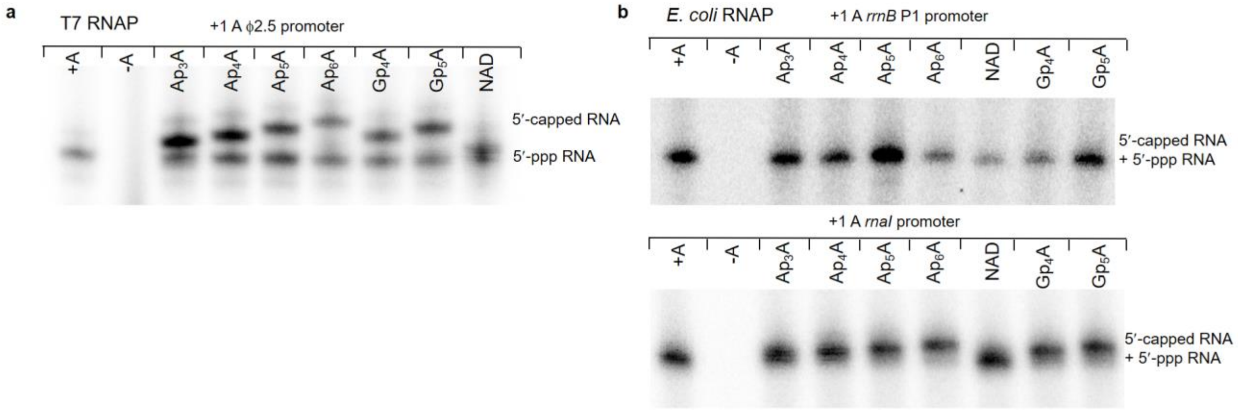

c

+1 A ϕ2.5 promoter

conc. of cap [mM]	**Ap$_3$A-RNA**	**Ap$_4$A-RNA**	**Ap$_5$A-RNA**	**Ap$_6$A-RNA**	**NAD**
**0.2**	16 ± 3.6	16 ± 2.9	18 ± 1.7	7 ± 1.7	13 ± 1.4
**0.4**	25 ± 2.6	23 ± 0.9	22 ± 2.5	15 ± 0.8	21 ± 2.5
**1.0**	37 ± 2.9	38 ± 3.1	38 ± 4.6	27 ± 1.7	33 ± 2.4
**1.6**	51 ± 6.0	48 ± 1.9	43 ± 2.9	36 ± 1.2	42 ± 2.6

d

+1 G ϕ6.5 promoter

conc. of cap [mM]	**Gp$_4$G-RNA**	**Ap$_4$G-RNA**	**Ap$_5$G-RNA**
**0.2**	22 ± 2.1	22 ± 0.9	21 ± 1.6
**0.4**	28 ± 2.1	30 ± 2.1	29 ± 3.7
**1.0**	44 ± 2.4	46 ± 2.9	42 ± 3.3
**1.6**	53 ± 2.1	55 ± 2.2	46 ± 5.2

Supplementary Figure 1: In vitro transcription with T7 and *E. coli* RNAP. **a,** *In vitro* transcription with T7 RNAP, template containing promoter Aϕ2.5 and various Np$_n$Ns or NAD as NCIN. **b,** *In vitro* transcription with *E. coli* RNAP, template containing promoter A *rrnB* P1 or *rnaI* and various Np$_n$Ns or NAD as NCIN. **c-d,** Tables of the amount of capped RNA (in %) compared to the uncapped 5'-ppp RNA prepared by *in vitro* transcription with T7 RNAP in the presence of constant amount of ATP (1 mM) or GTP (1 mM) and increasing amount (0.2-1.6 mM) of Np$_n$Ns or NAD (caps) in the solution. The percentage was determined after PAGE analysis of the *in vitro* transcription using 35A or 35G template. The RNA was labelled by α-^{32}P GTP. The average values and their standard deviations were calculated from triplicates.

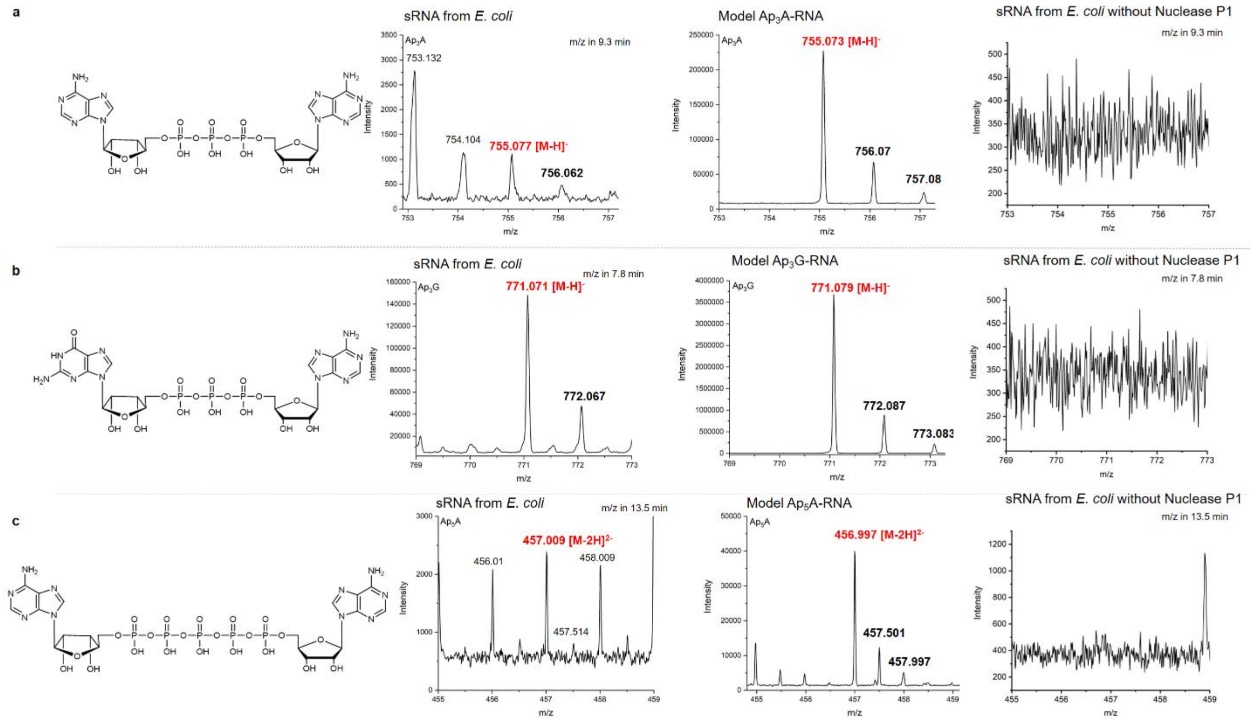

Supplementary Figure 2: LC-MS detection of naturally occurring Np$_n$N-RNA in *E. coli.* From the top; structures of Ap$_3$A (**a**), Ap$_3$G (**b**) and Ap$_5$A (**c**) and comparison of observed MS spectra in RNA from *E. coli*, synthetic standards and negative control (RNA from *E. coli* without Nuclease P1 treatment) (experiments were performed in biological triplicates).

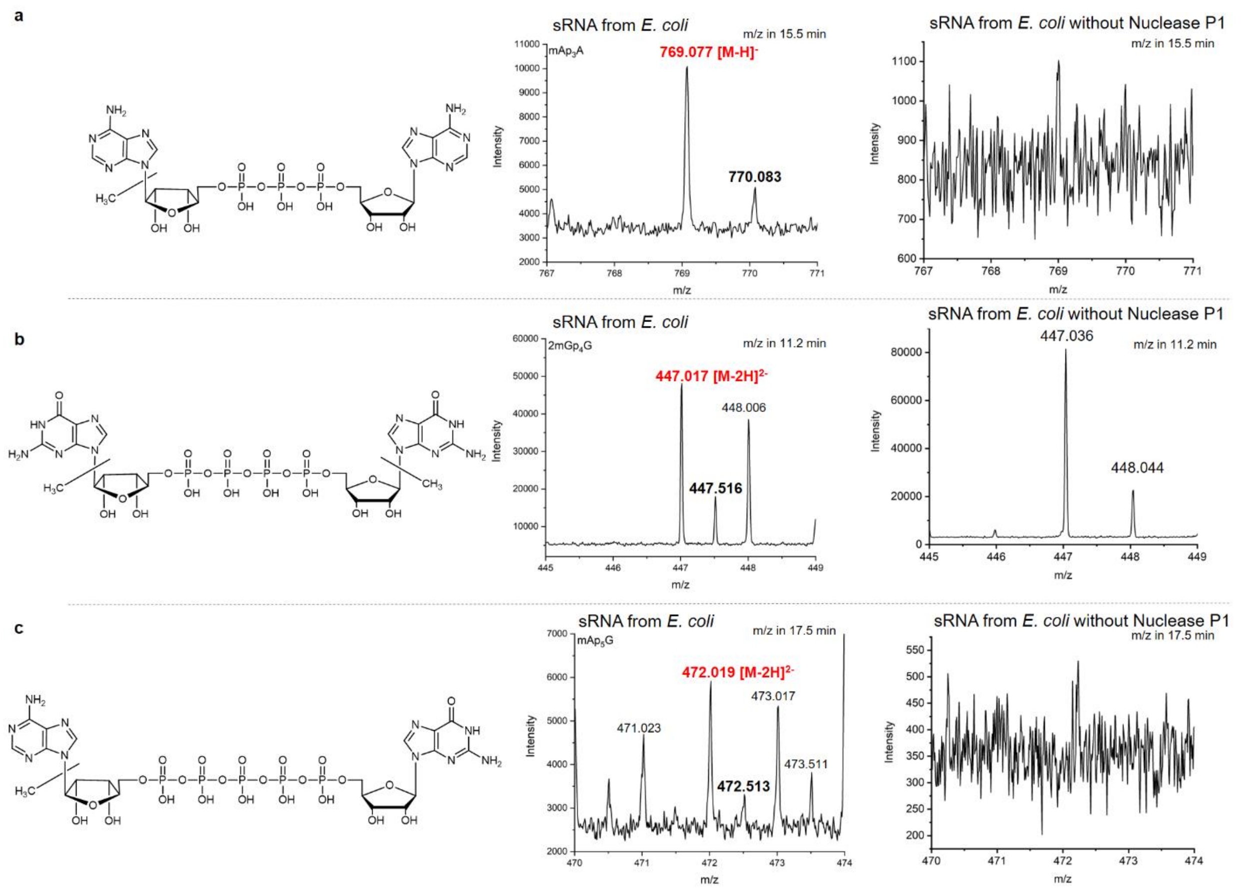

Supplementary Figure 3: LC-MS detection of naturally occurring Np$_n$N-RNA in *E. coli.* From the top; structures of mAp$_3$A (**a**), 2mGp$_4$G (**b**) and mAp$_5$G (**c**) and comparison of observed MS spectra in RNA from *E. coli* and negative control (RNA from *E. coli* without Nuclease P1 treatment) (experiments were performed in biological triplicates).

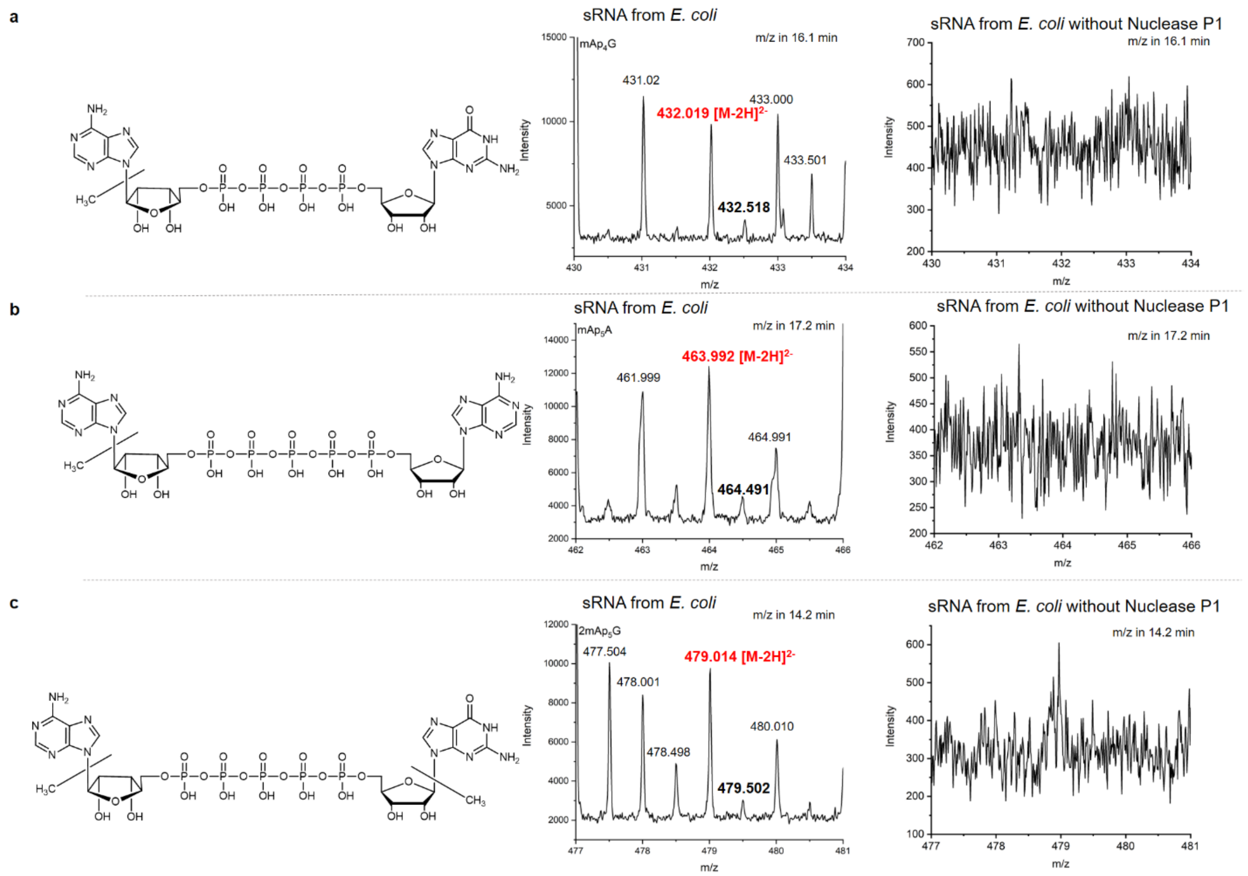

Supplementary Figure 4: LC-MS detection of naturally occurring Np$_n$N-RNA in *E. coli.* From the top; structures of mAp$_4$G (**a**), mAp$_5$A (**b**) and 2mAp$_5$G (**c**) and comparison of observed MS spectra in RNA from *E. coli* and negative control (RNA from *E. coli* without Nuclease P1 treatment) (experiments were performed in biological triplicates).

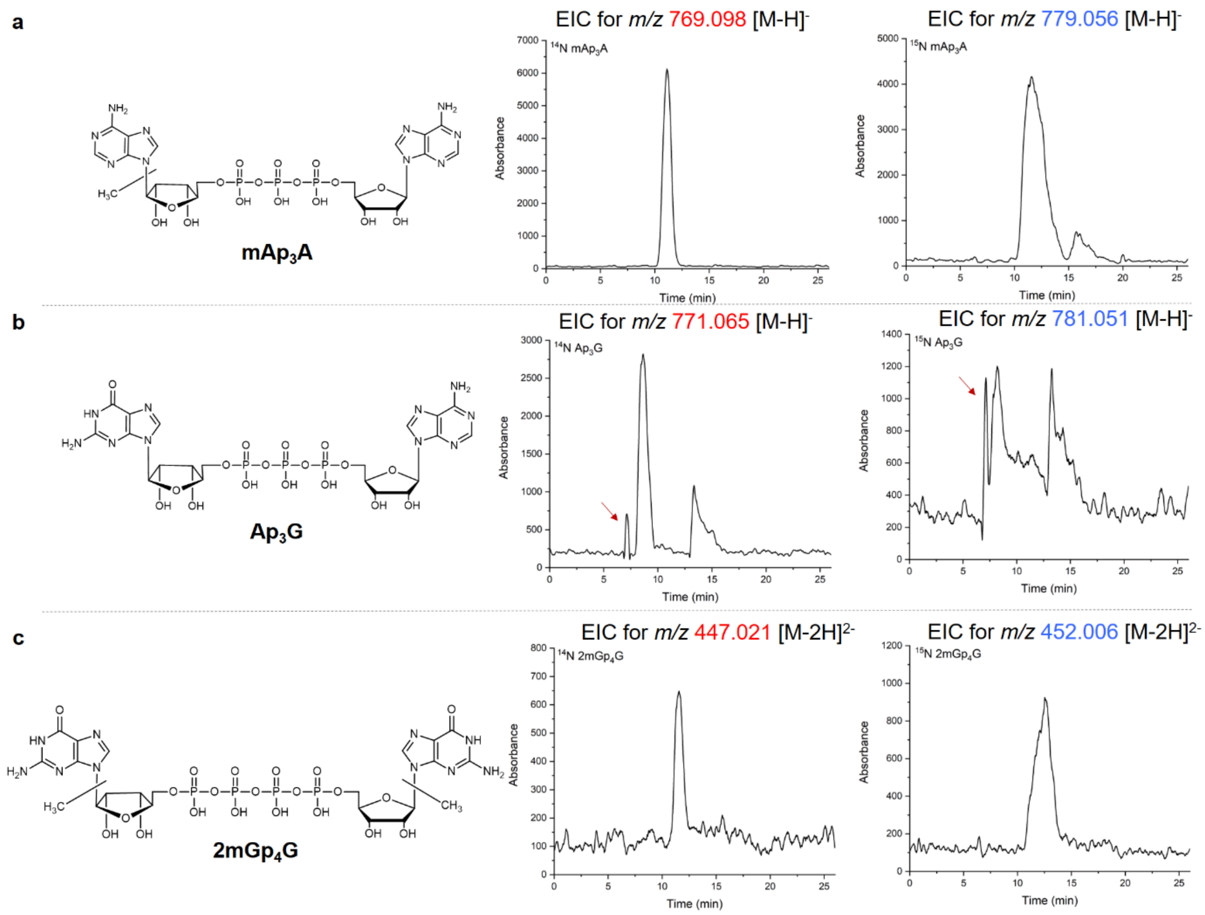

Supplementary Figure 5: Structures of different RNA caps and Extracted Ion Chromatograms (EIC) for various *m/z* in RNA from *E. coli* growth in minimal media containing ^{14}N or ^{15}N of methyl-Ap_3A (**a,** *m/z* 769.098 in ^{14}N corresponds to *m/z* 779.056 in ^{15}N in 11 min retention time), Ap_3G (**b,** *m/z* 771.065 in ^{14}N corresponds to *m/z* 781.051 in ^{15}N in 7 min retention time), dimethyl-Gp_4G (**c,** *m/z* 447.021 in ^{14}N was corresponds to *m/z* 452.006 in ^{15}N in 12 min retention time) (experiments were performed in biological triplicates).

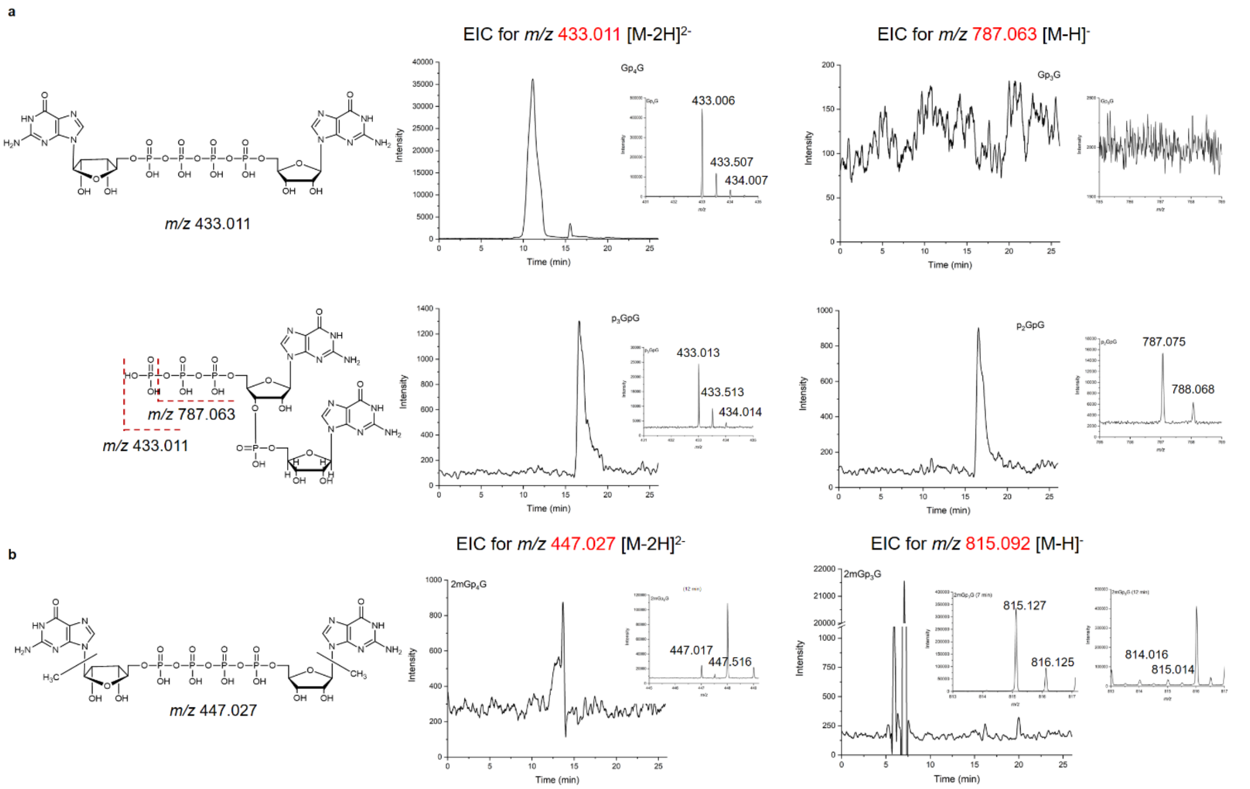

Supplementary Figure 6: Confirmation of 5ʹ-5ʹʹpolyphosphate chain. **a,** Fragmentation of synthetic standards. Structure and LC-MS analysis of model Gp_4G and p_3GpG. Extracted Ion Chromatograms (EIC) for *m/z* 433.011 (Gp_4G and p_3GpG, $[M\text{-}2H]^{2-}$) (middle) and for *m/z* 787.063 (Gp_3G and p_2GpG, $[M\text{-}H]^{-}$) (right). Molecule p_3GpG with external polyphosphate chain undergoes the cleavage under the ionization conditions and *m/z* 787.063 is observed. While Gp_4G with internal polyphosphate chain stays under the same conditions intact. **b,** Structure and LC-MS analysis of detected $2mGp_4G$. EIC for *m/z* 447.027 ($2mGp_4G$. $[M\text{-}2H]^{2-}$) (middle) and for *m/z* 815.092 ($2mGp_3G$, $[M\text{-}H]^{-}$) (right). EIC is comparable with EIC of model Gp_4G confirming the internal polyphosphate chain (experiments were performed in biological triplicates).

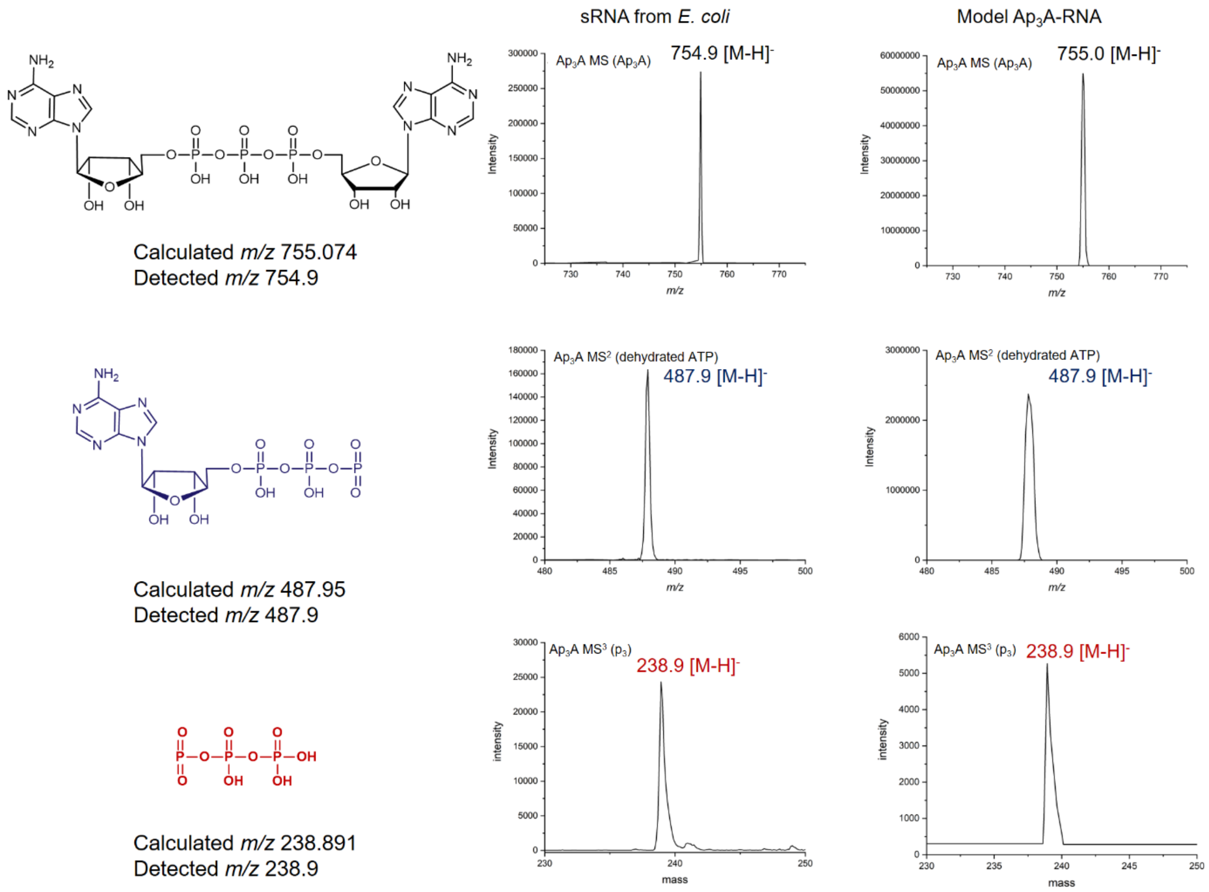

Supplementary Figure 7: The fragmentation behaviour of detected Ap_3A. Ap_3A ion was fragmented and peak with *m/z* 487.9 (dehydrated ATP, $[M\text{-}H]^-$) was selected for further fragmentation (MS^3). The *m/z* 238.9 $[M\text{-}H]^-$ corresponds to the intact polyphosphate chain. The same behaviour was observed for Ap_3A standard (right) as for Ap_3A coming from the analysed sample from RNA of *E. coli* (middle) (experiments were performed in biological triplicates).

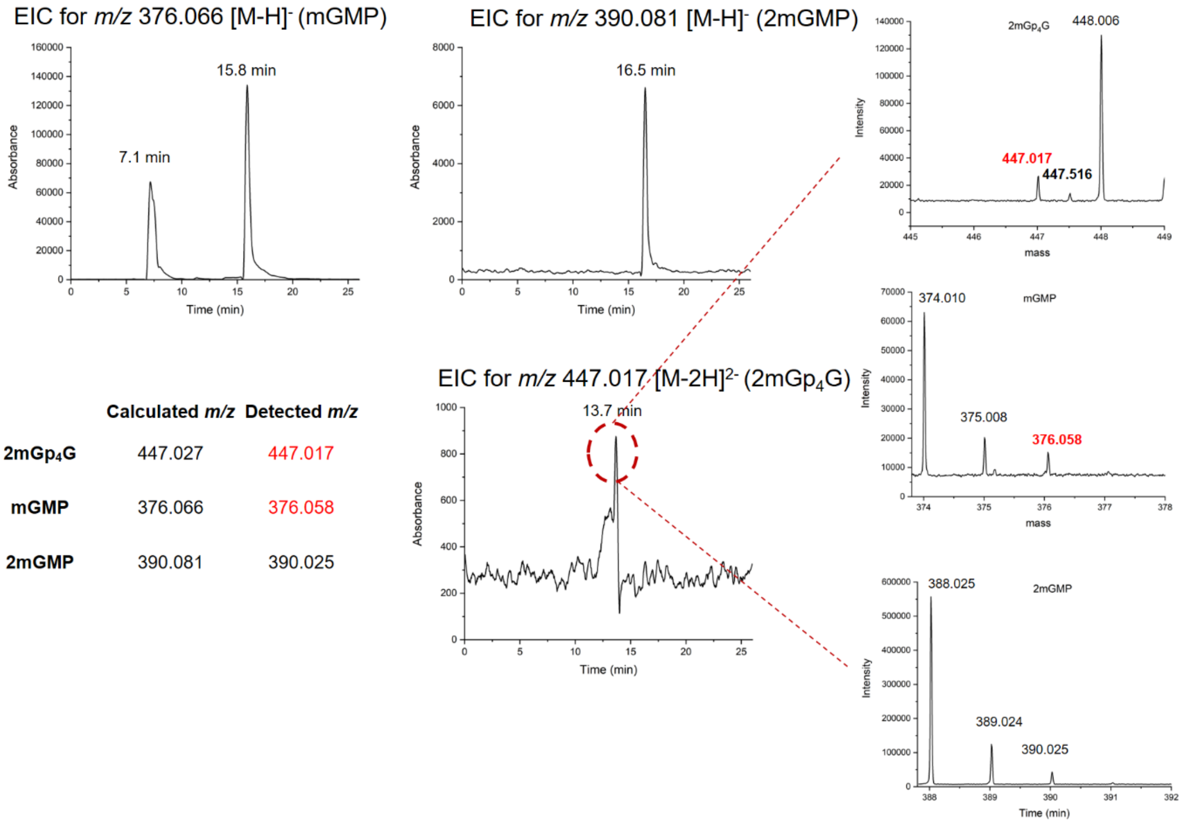

	Calculated *m/z*	Detected *m/z*
**2mGp$_4$G**	447.027	447.017
**mGMP**	376.066	376.058
**2mGMP**	390.081	390.025

Supplementary Figure 8: Fragmentation pattern of 2mGp$_4$G. Distribution of methyl groups in detected 2mGp$_4$G. Extracted Ion Chromatograms (EIC) of fragments of *m/z* 376.066 (mGMP, $[M-H]^-$) and *m/z* 390.081 (2mGMP, $[M-H]^-$) in sRNA from *E. coli*. EIC for *m/z* 447.017 (2mGp$_4$G, $[M-2H]^{2-}$) and MS spectra for 13.7 min. The MS spectra confirmed the presence of monomethylated GMP (*m/z* 376.058, $[M-H]^-$) as fragment coming from 2mGp$_4$G. The calculated *m/z* 390.081 corresponding to dimethylated GMP was not detected in time 13.7 min. Detected ion of m/z 390.025 is not 2mGMP since it is *isotopologue* of *m/z* 388.025 and the error of the measurement is more than 140 ppm. The average error of the measurement is 25 ppm (experiments were performed in biological triplicates).

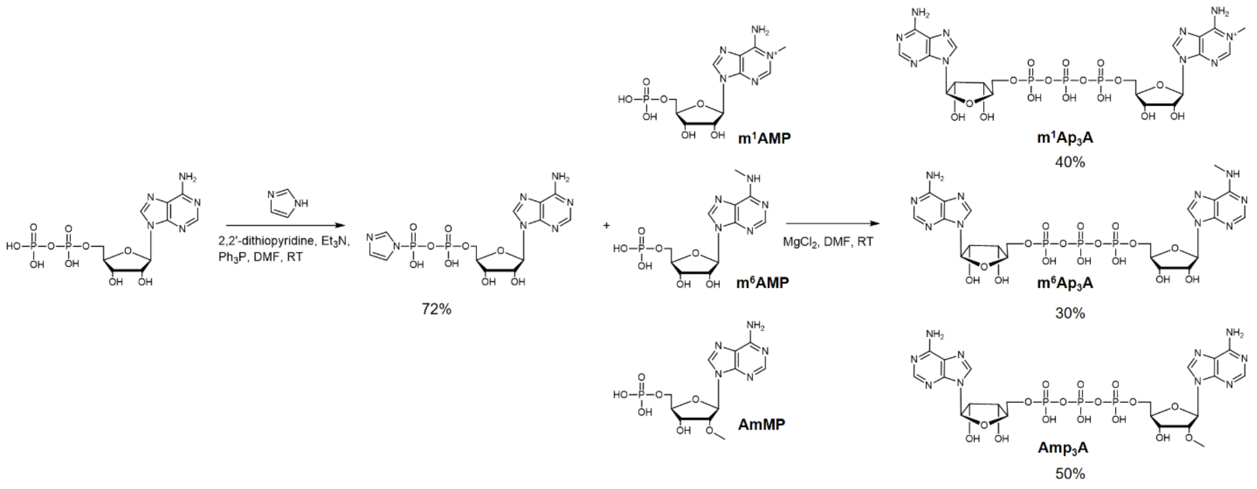

Supplementary Figure 9: Scheme of synthesis of methylated Ap_3A. In the first step, ADP (adenosine diphosphate) was converted to adenosine 5'-diphosphate imidazolide in 72% yield. In the second step, it was coupled to mono-methylated AMP (adenosine monophosphate) resulting in one of the mono-methylated Ap_3A: m^1Ap_3A, m^6Ap_3A and Amp_3A.

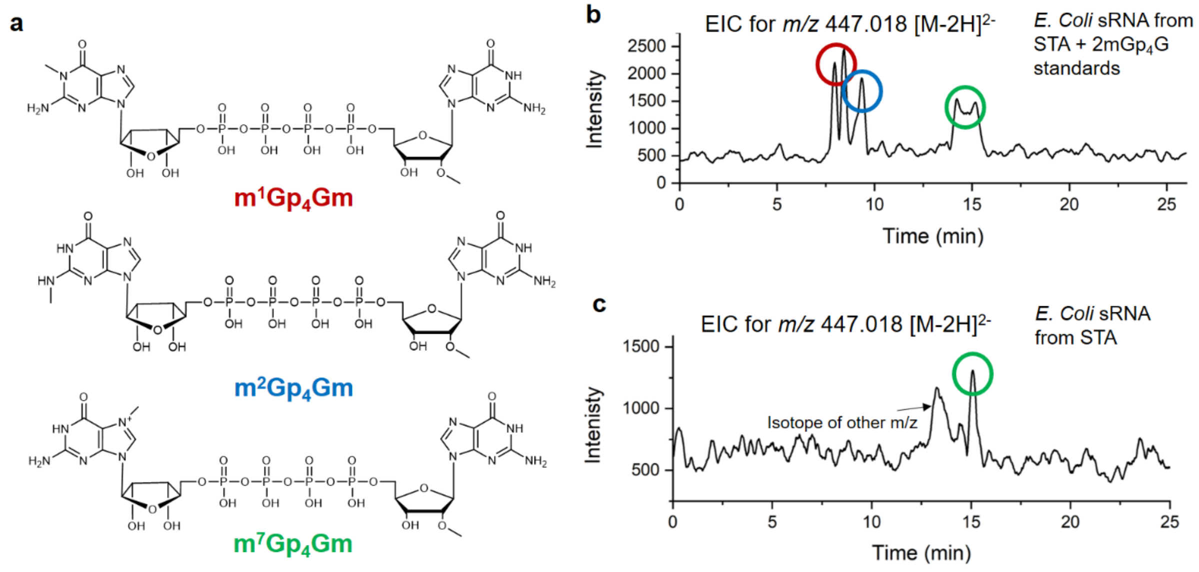

Supplementary Figure 10: **a,** The chemical structures of dimethylated Gp_4G used as standards for the structure determination of detected dimethylated Gp_4G in *E. coli* RNA. **b,** Extracted Ion Chromatogram (EIC) for m/z 447.018 ($2mGp_4G$) in sRNA sample from STA of *E. coli* with spiked standards. **c,** Extracted Ion Chromatogram (EIC) for m/z 447.018 ($2mGp_4G$) in sRNA sample from STA of *E. coli* without any spiked standards (experiments were performed in biological triplicates).

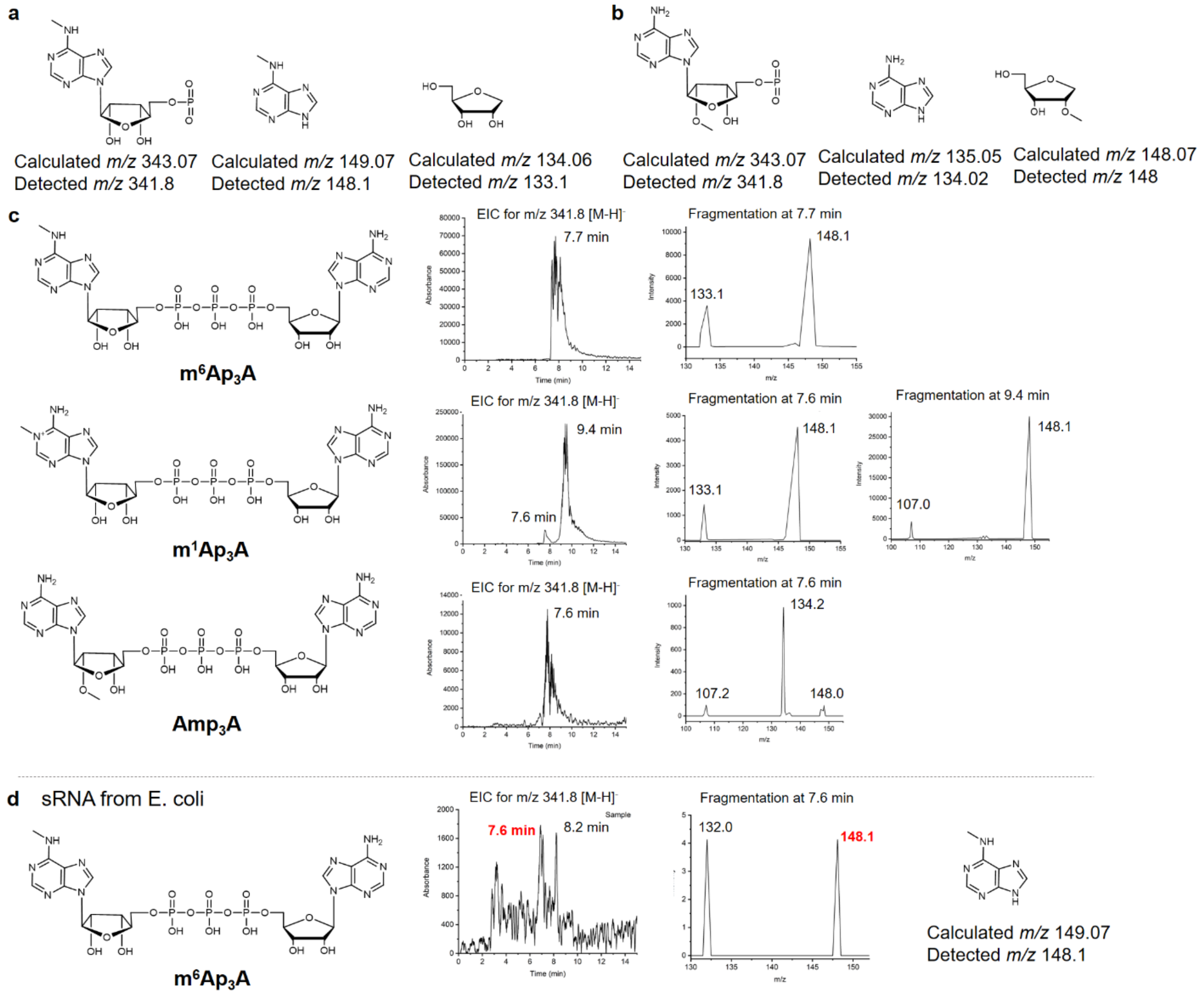

Supplementary Figure 11: Fragmentation study of mAp$_3$A using QTRAP (low resolution instrument). ***a*,** Main calculated and detected fragments of m^{6}Ap$_3$A or m^{1}Ap$_3$A. **b*,*** Main calculated and detected fragments of Amp$_3$A. **c,** LC-MS analysis and fragmentation of synthetic standards m^{6}Ap$_3$A, m^{1}Ap$_3$A and Amp$_3$A. m^{6}Ap$_3$A, and Amp$_3$A have same retention time between 7.6 and 7.7 min. The fragmentation of methylated AMP (*m/z* 341.8) leads to fragment 148.1 in case of m^{6}Ap$_3$A and 134.2 in case of Amp$_3$A. m^{1}Ap$_3$A has retention time at 9.4 min, but it partially converts to m^{6}Ap$_3$A under basic measurement conditions. The further fragmentation of methylated AMP (*m/z* 341.8) from m^{1}Ap$_3$A leads to main fragment 148.1. **d*,*** LC-MS analysis and fragmentation of digested sRNA from *E. coli*. The retention time of the main fragment 341.8 is 7.6 min, corresponding to m^{6}Ap$_3$A or Amp$_3$A. Further fragmentation leads to main fragment *m/z* 148.1 confirming the methylated adenine and thus m^{6}Ap$_3$A (all experiments were performed in biological triplicates).

.

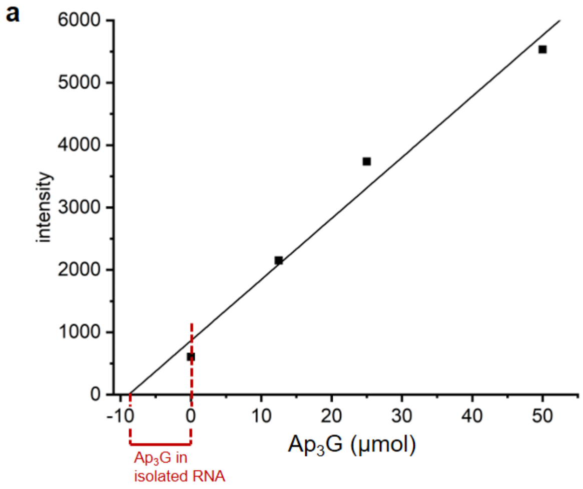

b

Caps	Absolute amount of cap (fmol/µgRNA)
**Ap_3A**	61 ± 16
**Ap_3G**	83 ± 29
**m^7Gp_4Gm**	1197 ± 109
**Ap_5A**	98 ± 21
**NAD**	1896 ± 75
**CoA**	41 ± 17

Supplementary Figure 12: **a,** Example of regression curve used for absolute quantification of Ap_3G in sRNA isolated from *E. coli.* 12.5, 25 and 50 µmol of standard Ap_3G was added to isolated RNA and analysed by LC-MS. ***b,*** Absolute quantification of non-canonical RNA caps in *E. coli* sRNA isolated in STA (all experiments were performed in biological triplicates).

.

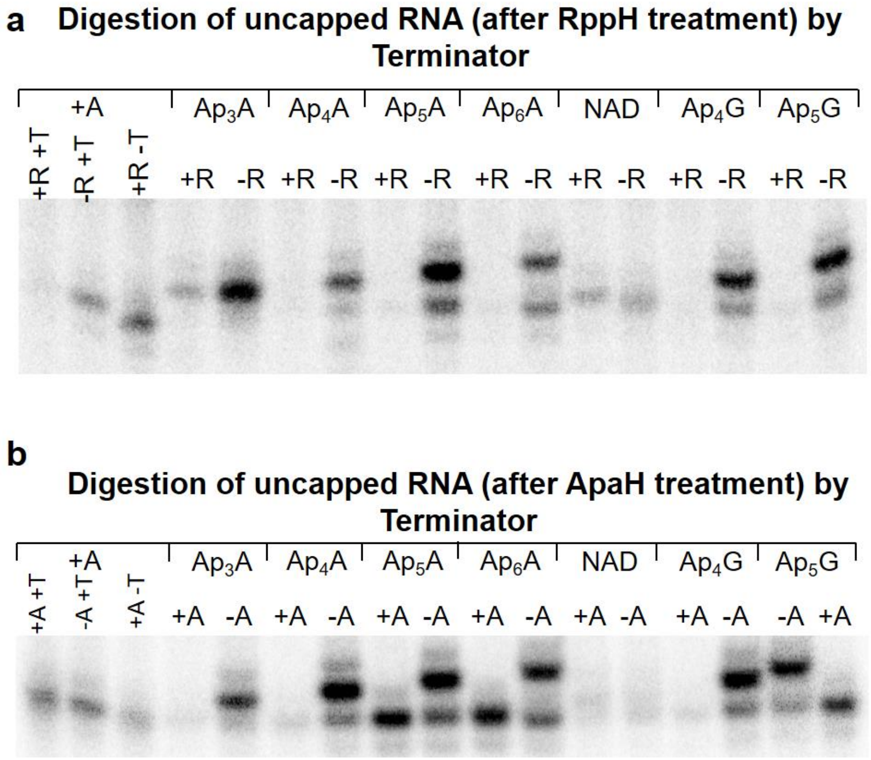

Supplementary Figure 13: PAGE (12%) analysis of *in vitro* transcribed RNA (35nt) after RppH (**a**) and ApaH (**b**) cleavage followed by terminator treatement. Terminator degrades all the 5'-p RNA (all experiments were performed in triplicates).

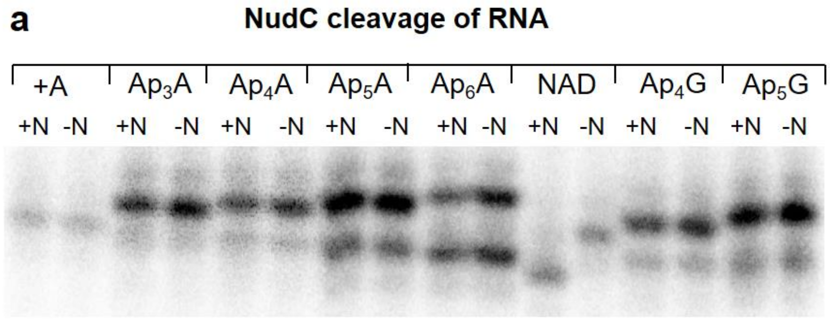

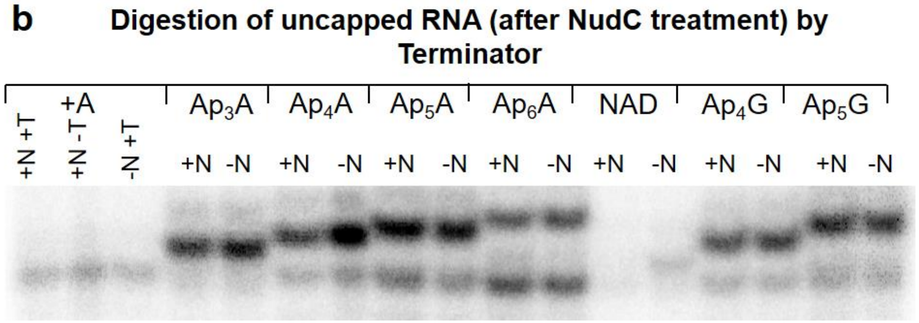

Supplementary Figure 14: PAGE (12%) analysis of *in vitro* transcribed RNA (35nt) **a,** treated with NudC (+N) or without (-N) **b,** and followed by terminator treatement (all experiments were performed in triplicates).

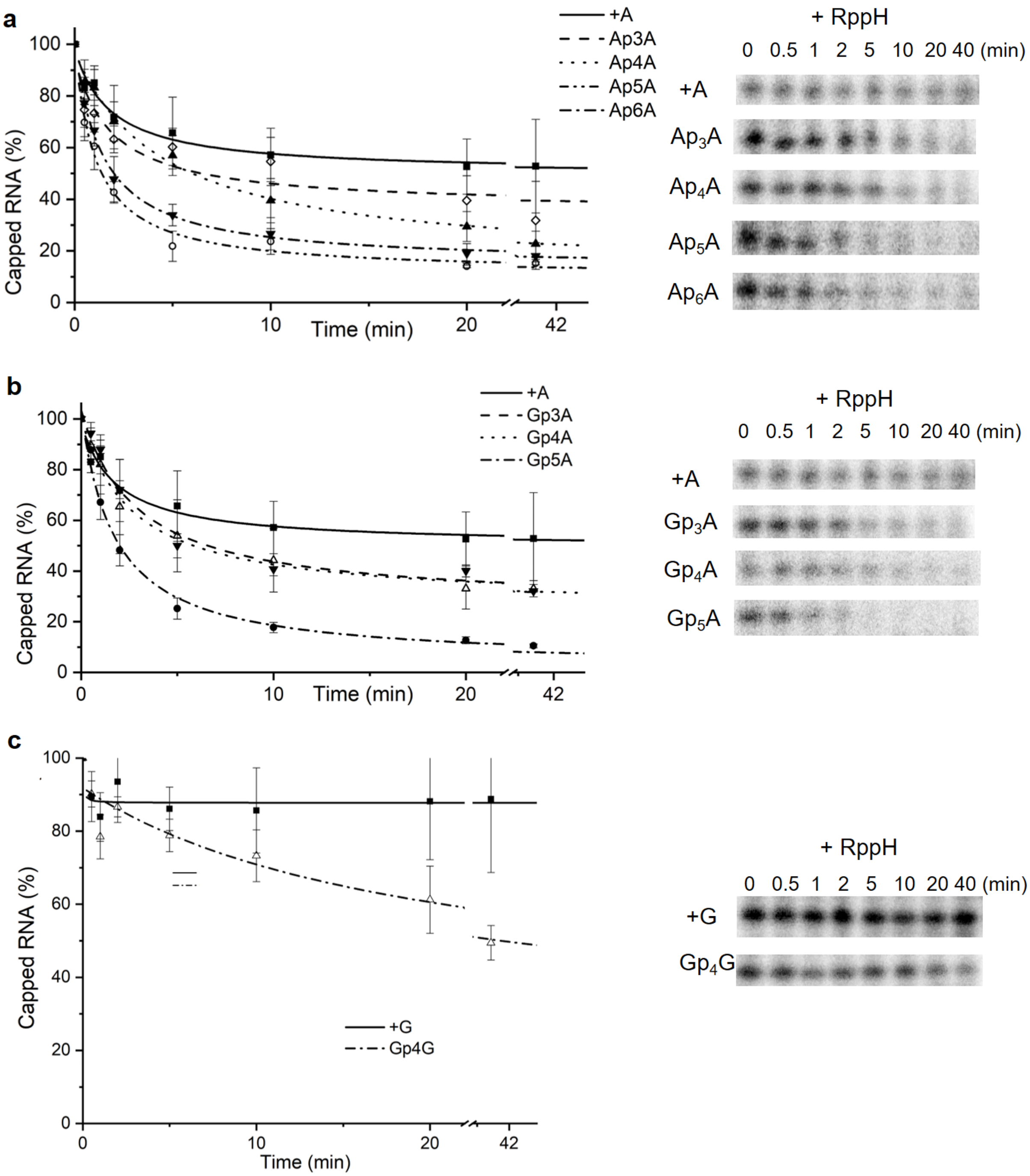

Supplementary Figure 15: RppH cleavage kinetic study. ***a,*** Kinetic studies of RppH cleavage of Ap$_n$A-capped RNA in comparison with 5’-pppA RNA stopped after 30 s, 1, 2, 5, 10, 20 and 40 min and analysed by PAGE. **b,** Kinetic studies of RppH cleavage of Gp$_n$A-capped RNA in comparisom with 5’-pppA RNA stopped after 30 s, 1, 2, 5, 10, 20 and 40 min and analysed by PAGE. **c,** Kinetic studies of RppH cleavage of Gp_4G-capped in comparison with 5’-pppG RNA stopped after 30 s, 1, 2, 5, 10, 20 and 40 min and analysed by PAGE. All experiments were performed in triplicates and calculated as average values. Error bars indicate standard deviations. Source data are provided in the Source Data file.

.

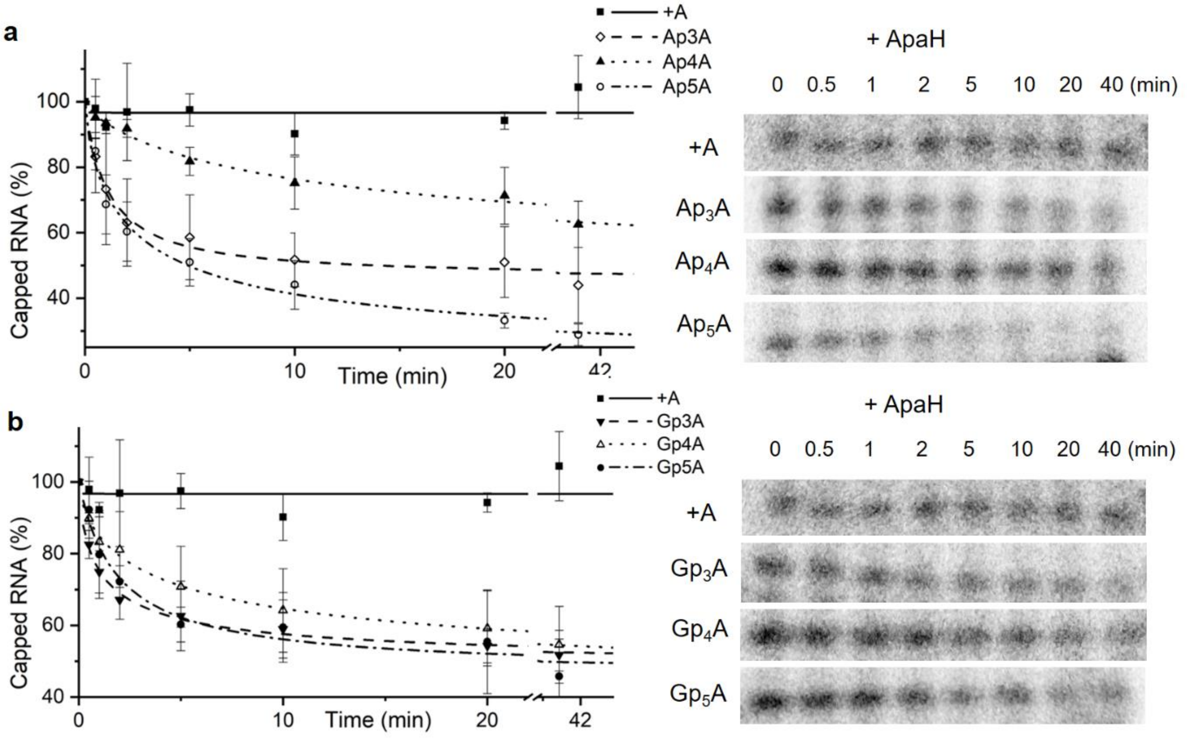

Supplementary Figure 16: ApaH cleavage kinetic study. ***a,*** Kinetic studies of ApaH cleavage of Ap_nA-capped RNA in comparison with 5’-pppA RNA stopped after 30 s, 1, 2, 5, 10, 20 and 40 min and analysed by PAGE. **b,** Kinetic studies of ApaH cleavage of Gp_nA-capped RNA in comparisom with 5’-pppA RNA stopped after 30 s, 1, 2, 5, 10, 20 and 40 min and analysed by PAGE. All experiments were performed in triplicates and calculated as average values. Error bars indicate standard deviations. Source data are provided in the Source Data file.

.

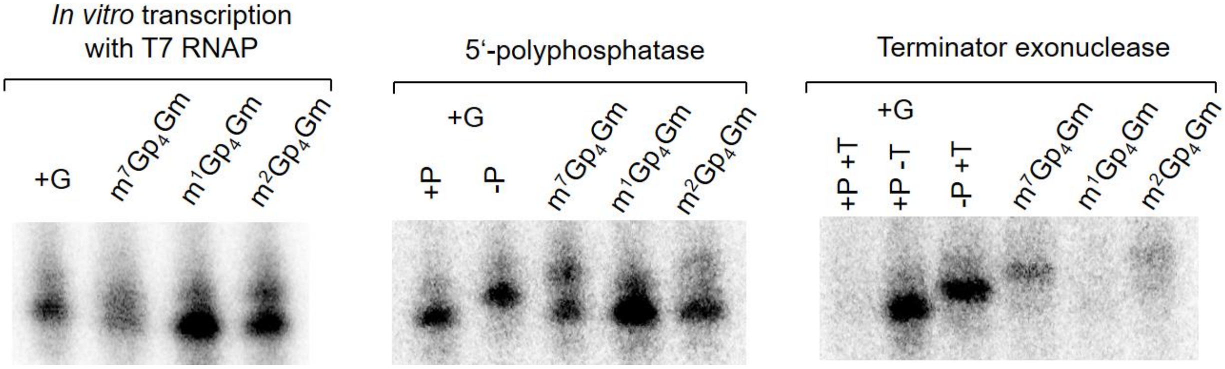

Supplementary Figure 17: Polyacrylamide gel electrophoretic (PAGE - 12%) analysis of the (α-^{32}P GTP labelled) *in vitro* transcription products (35 nt) with T7 RNAP and m^7Gp_4Gm, m^1Gp_4Gm and m^2Gp_4Gm followed by P and/or T treatment (all experiments were performed in triplicates).

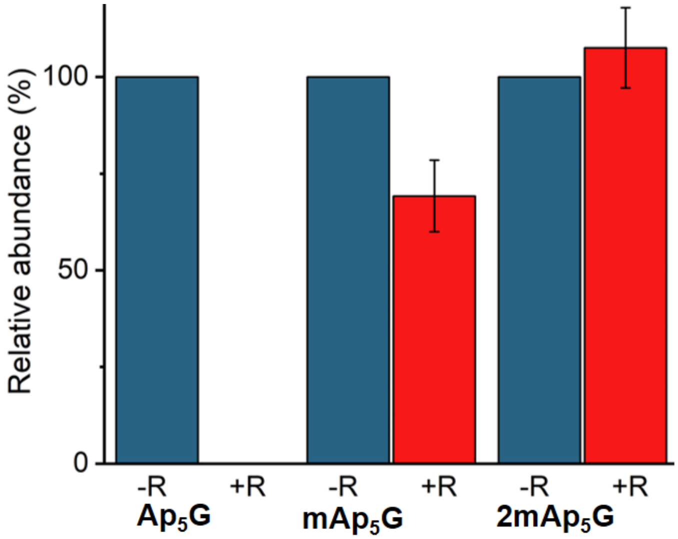

Supplementary Figure 18: Relative abundance of non-methylated A_5G (left), mAp_5G (middle) and $2mAp_5G$ (right)-RNA as derived from EIC in the sRNA fraction spiked with Ap_5G-RNA before (blue), after 1 h RppH treatment (red). All experiments were performed in triplicates and calculated as average values. Error bars indicate standard deviations. Source data are provided in the Source Data file.

Absolute Expression Levels (transcripts per million) of mRNA in *Salmonella Typhimurium*

mRNA	Early exponential phase	Mid-exponential phase	Late exponential phase	Early stationary phase	Late stationary phase
ApaH	66	57	51	40	20
NudH	228	255	244	192	79

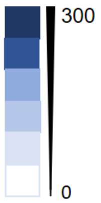

Supplementary Figure 19: The absolute expression levels of mRNA of ApaH and NudH in *Salmonella Typhimurium* in various stages of growth (http://bioinf.gen.tcd.ie/cgi-bin/salcom.pl?_HL).

REYES PR2182-1
1H NMR in D2O
26-08-19 RA
****

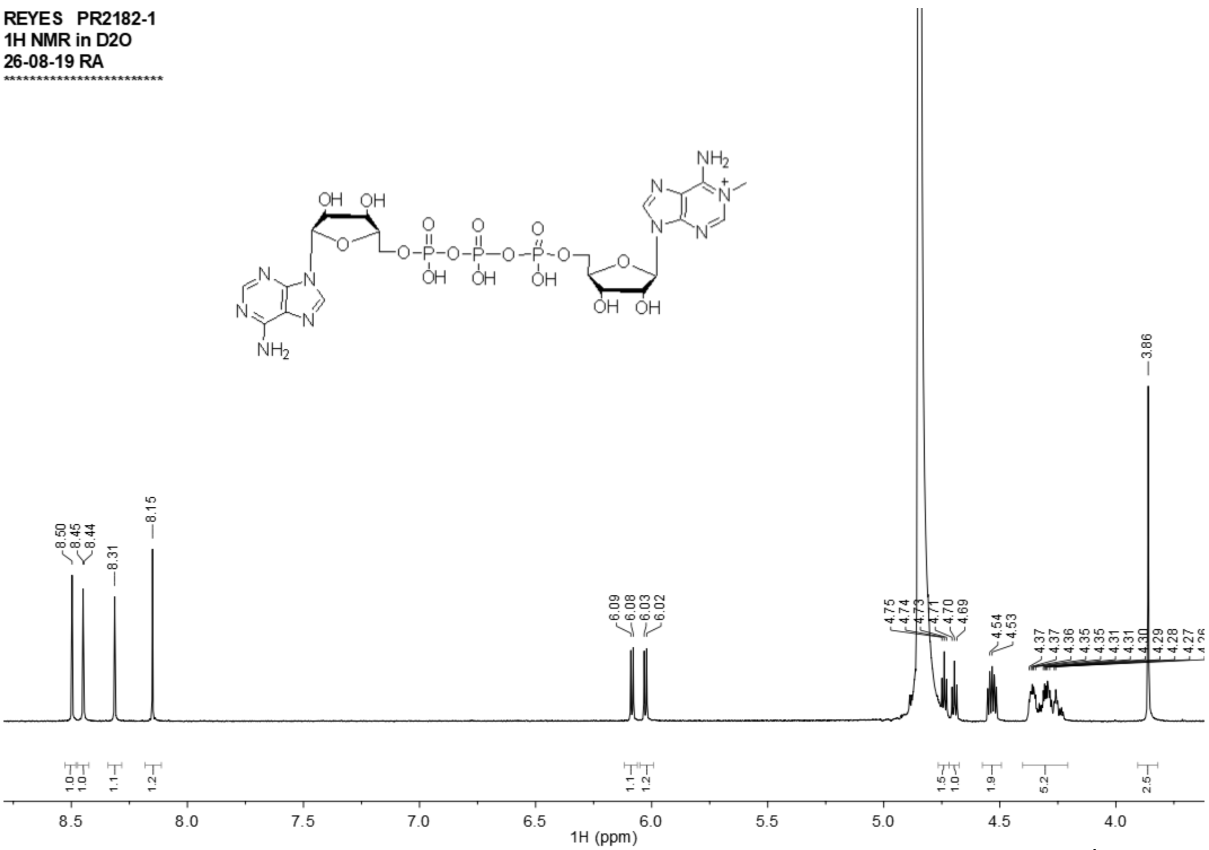

REYES PR2182-1
APT in D2O
26-08-19 RA
****

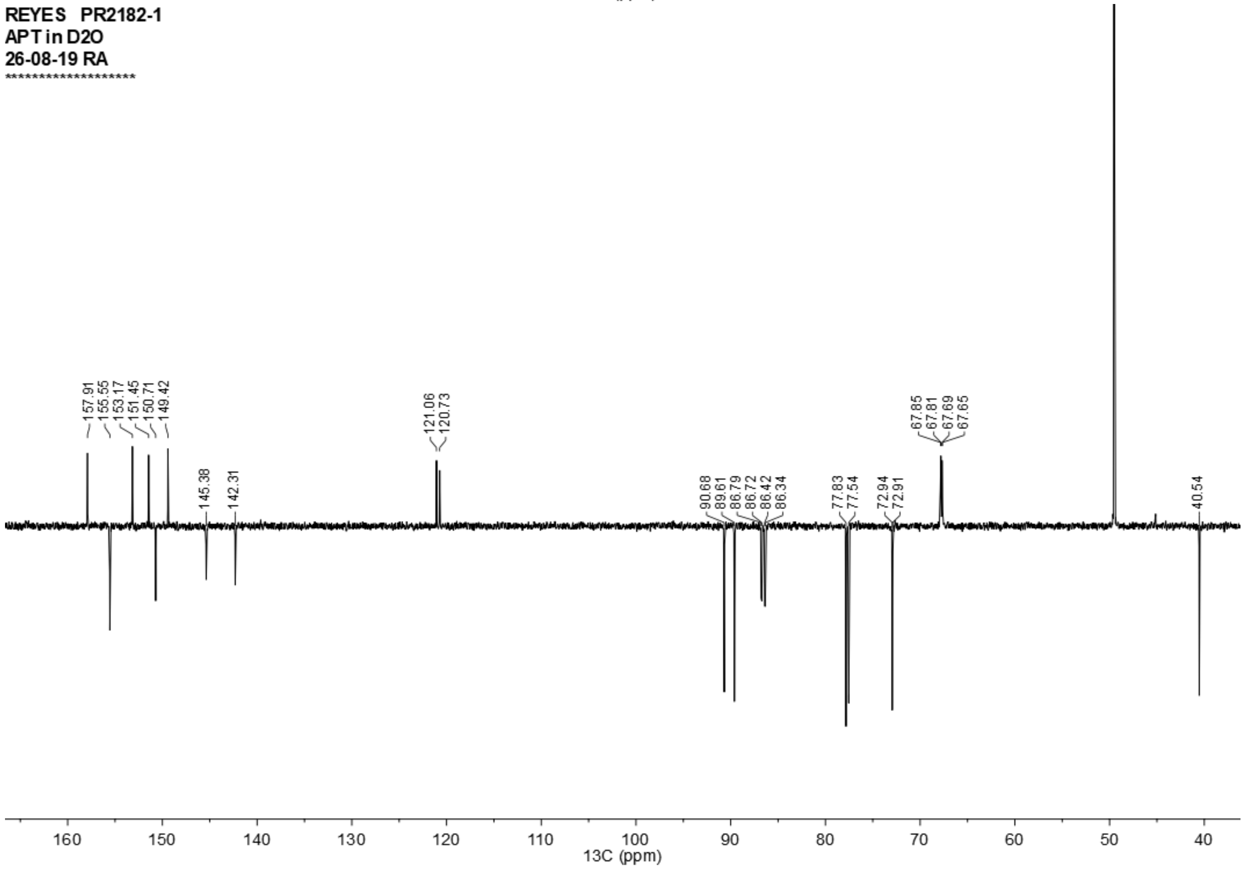

REYES PR2182-2
1H NMR in D2O
29-08-19 RA
****

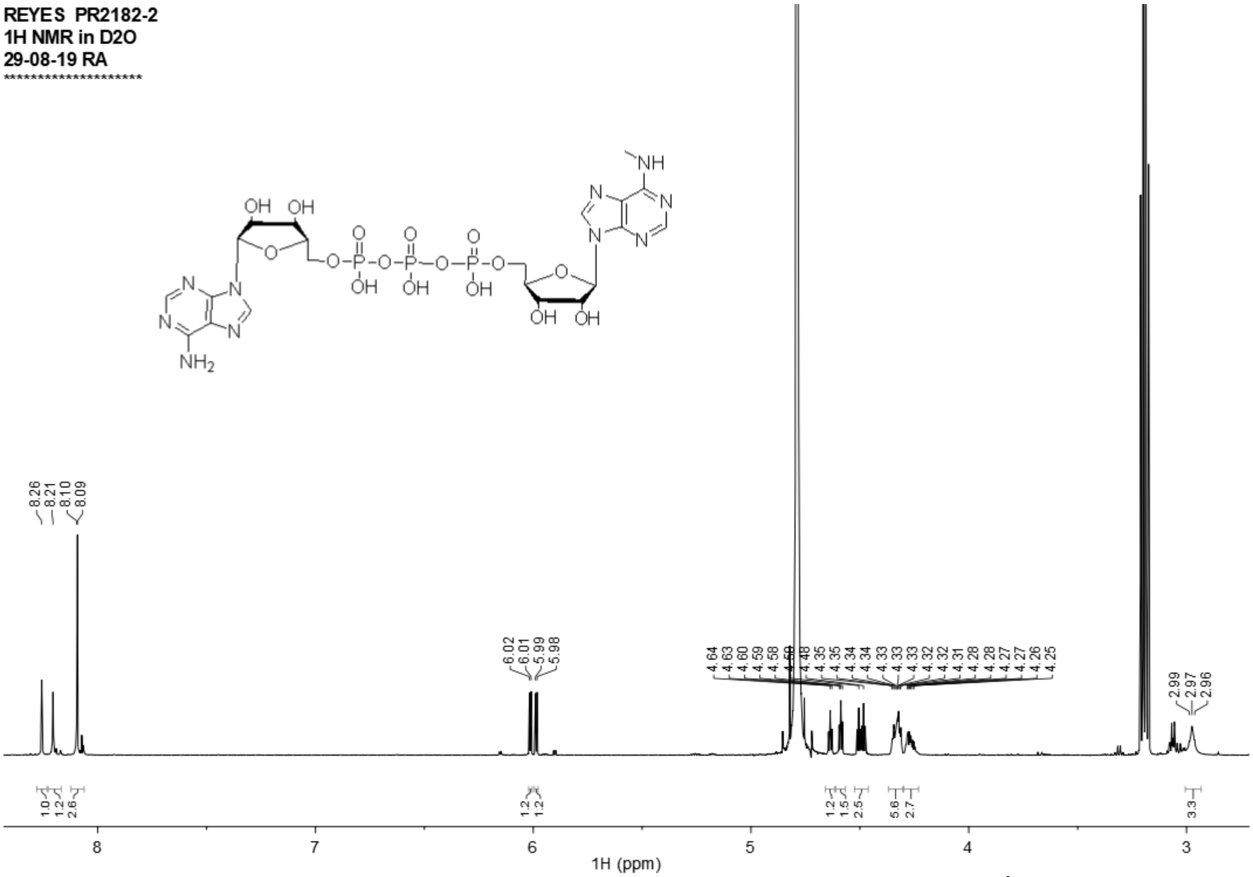

REYES PR2182-2
APT in D2O
28-08-19 RA
****

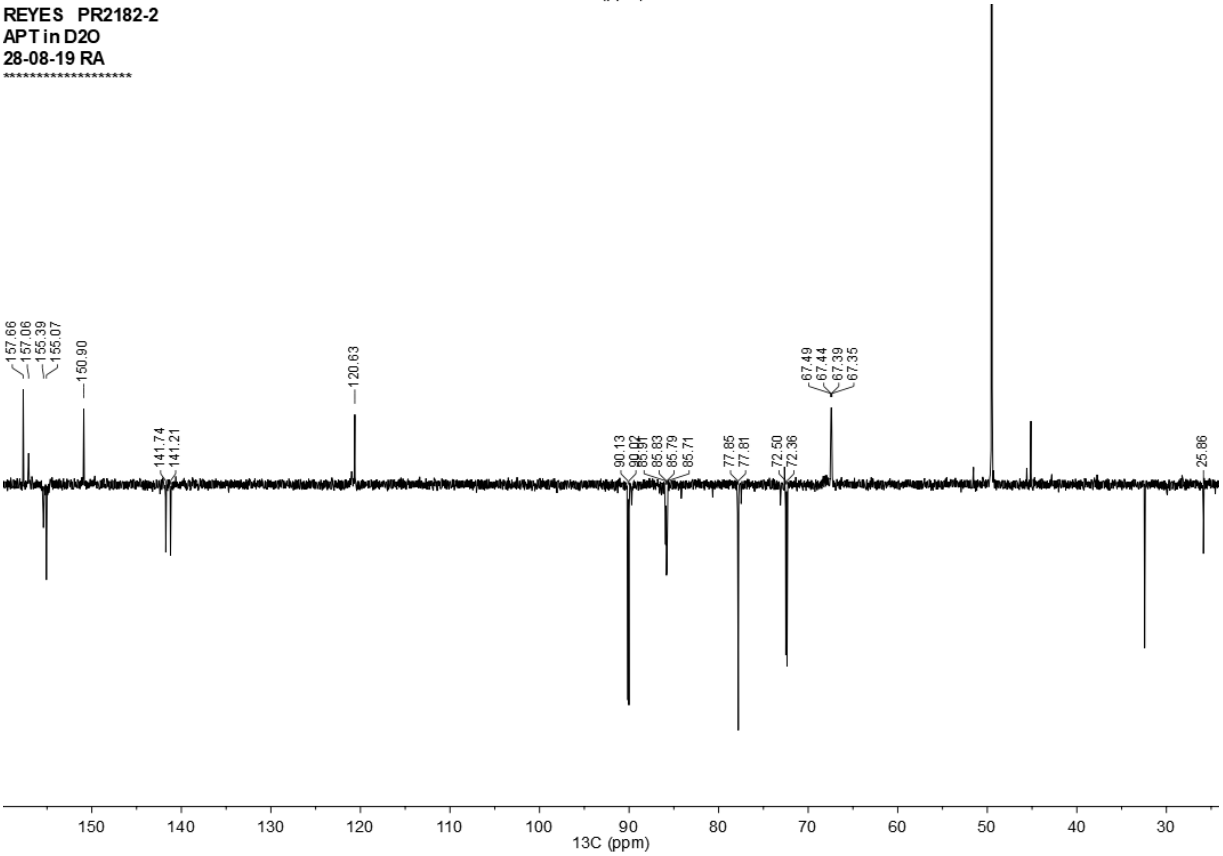

REYES PR2182-3
1H NMR in D2O
29-08-19 RA
****

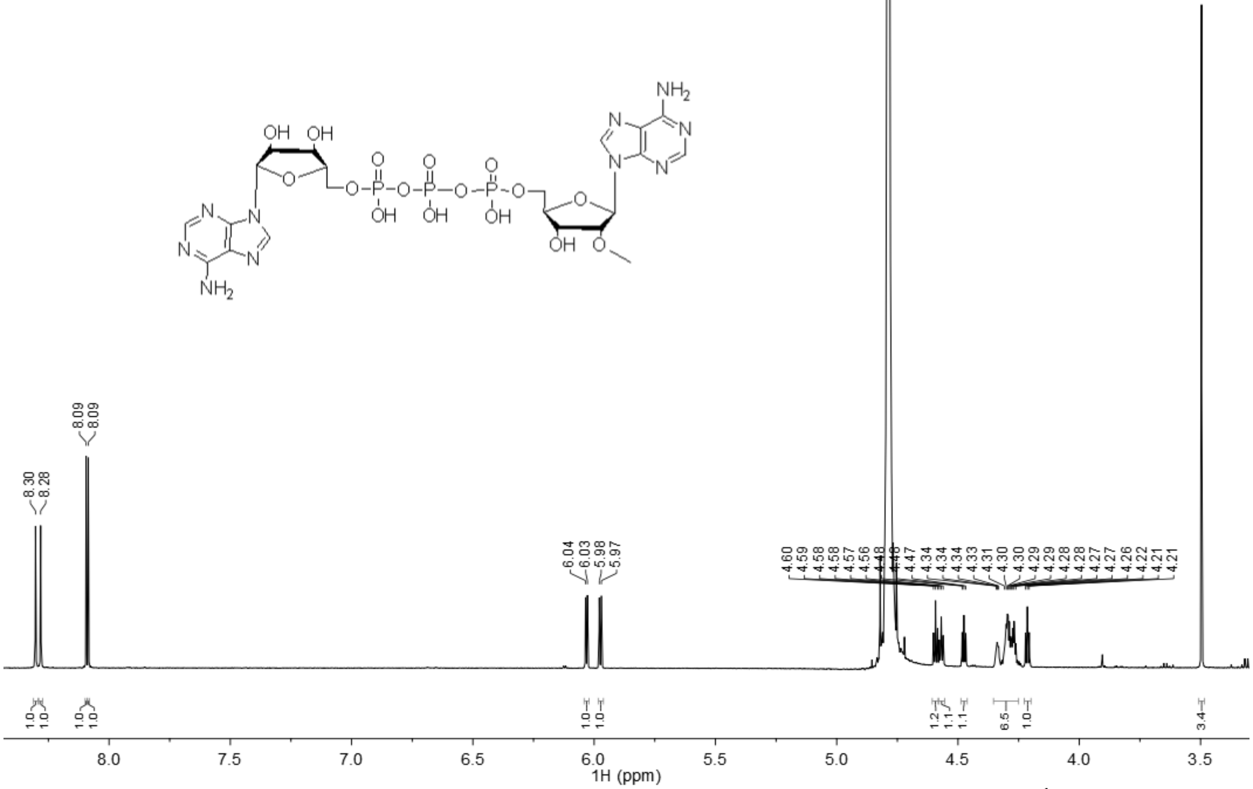

REYES PR2182-3
APT in D2O
29-08-19 RA
****

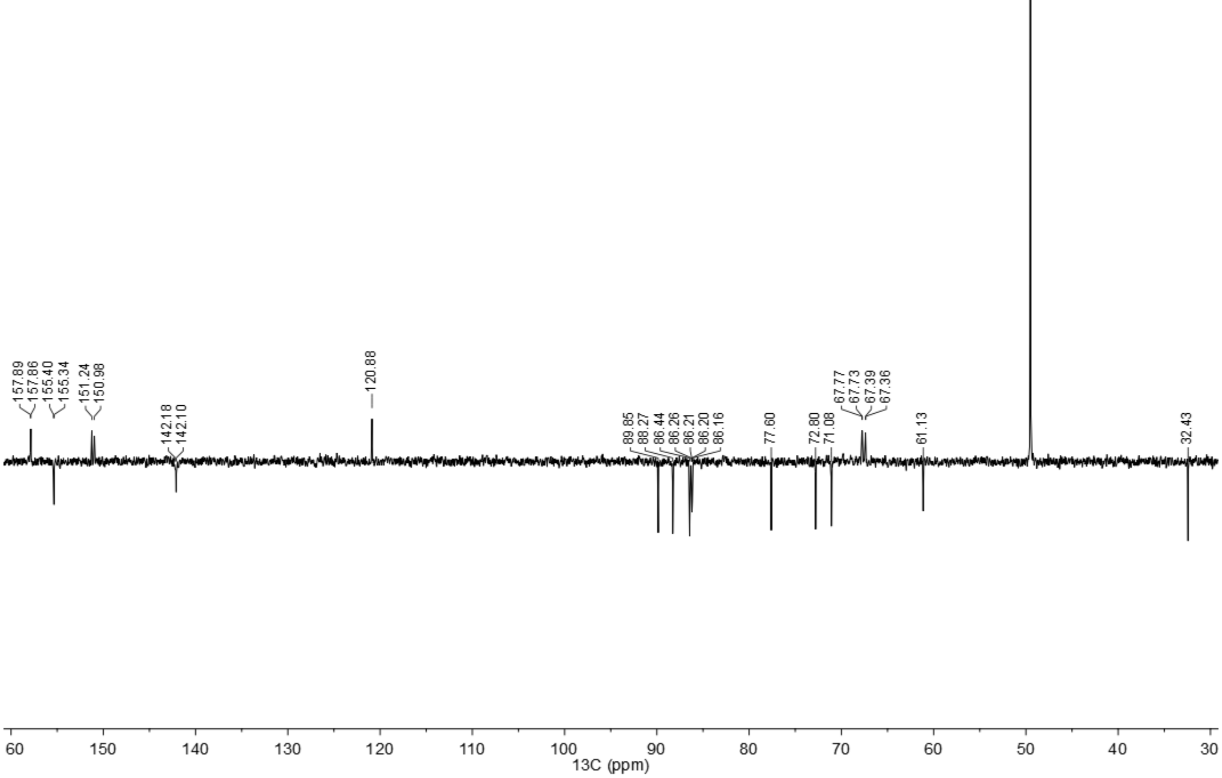

Supplementary Figure 20: ^{1}H and ^{13}C NMR spectra of mAp_3A standards.
